# IL-33 attenuates cardiac remodeling following myocardial infarction via inhibition of the p38 MAPK and NF-κB pathways

**DOI:** 10.3892/mmr.2022.12868

**Published:** 2022-10-04

**Authors:** Honglei Yin, Peicheng Li, Fudong Hu, Yakun Wang, Xiaoyan Chai, Yan Zhang

Mol Med Rep 9: 1834–1838, 2014; DOI: 10.3892/mmr.2014.2051

An interested reader of an article published in the journal *Circulation Research* [Krishnamurthy P, Rajasingh J, Lambers E, Qin G, Losordo DW and Kishore R: L-10 inhibits inflammation and attenuates left ventricular remodeling after myocardial infarction via activation of STAT3 and suppression of HuR. Circ Res 104: e9-18, 2008] drew to our attention that data featured in their paper had appeared subsequently in the abovementioned article by Yin *et al* in *Molecular Medicine Reports* in 2014. Specifically, Fig. 1 in the Mol Med Rep paper included the same histograms as those featured in Fig. 2 in the Circ Res paper; Fig. 2 in the Mol Med Rep paper contained data derived from Fig. 1 in the Circ Res paper; and Fig. 3, Fig. 4, Fig. 5 in both papers shared a substantial amount of the same data.

Following an internal investigation, the Journal was able to confirm that this accusation of plagiarism was well-founded. On those grounds, the Editor of Molecular Medicine Reports has decided to retract this paper. The authors were asked for an explanation to account for these concerns, but the Editorial Office did not receive a reply. The Editor deeply regrets the grievance that this matter has caused to the authors of the previously published article, and also any inconvenience caused to the readership of the Journal.

